# QUALITY IMPROVEMENT: IMPROVING ANTIBIOTIC STEWARDSHIP IN COMMUNITY-ACQUIRED PNEUMONIA

**DOI:** 10.51894/001c.123021

**Published:** 2024-08-30

**Authors:** Raagini Yedidi

**Affiliations:** 1 Internal Medicine Garden City Hospital

## 54

## INTRODUCTION

Withdrawing antibiotic treatment at the five-day mark in suitable inpatients with community-acquired pneumonia is recommended by the American Thoracic Society and the Infectious Disease Society of America. The goal of our intervention was to increase Resident confidence and knowledge regarding algorithmic management of community-acquired pneumonia.

## METHODS

A pre-intervention survey was conducted to assess Internal Medicine Resident pre-intervention confidence and knowledge regarding management of community-acquired pneumonia (CAP). A laminated flowsheet of preferred antibiotic regimens for uncomplicated CAP was distributed in the Resident workspace, and a one-hour lecture on this topic was held for all Internal Medicine Residents. Residents completed a post-intervention survey to determine whether confidence of uncomplicated CAP management and knowledge of preferred regimen selection had improved.

## RESULTS

Twenty-four Internal Medicine Residents were included for the intervention. The completion rate was 58.3% (14 Residents) for the pre-intervention survey and 87.5% (21 Residents) for the post-intervention survey. Residents rated their comfort level treating uncomplicated CAP from 1 to 5, 1 being uncomfortable and 5 being very comfortable. The average pre-intervention comfort level was 3.79, and the average post-intervention comfort level was 4.52 (p=0.0306). Before the intervention, 8 responses of 23 total (34.5%) were incorrectly selected pertaining to 7-day regimens and afterwards 5 of 37 total responses (13.5%) were incorrectly selected pertaining to 7-day regimens. Before the intervention, 84.62% (11) of respondents correctly identified ceftriaxone plus azithromycin/clarithromycin/doxycycline for 5 days and 23.08% (3) correctly identified ampicillin + sulbactam plus azithromycin/clarithromycin/doxycycline for 5 days of treatment as preferred regimens for uncomplicated CAP. Afterwards, 90.48% of respondents (19) correctly identified the first regimen and 47.62% of respondents (10) correctly identified the second.

## CONCLUSIONS

The intervention was successful in increasing Resident confidence in treating uncomplicated CAP. The intervention also decreased the incorrect response rate of 7-day regimens for the treatment of uncomplicated CAP, representing increased Resident awareness of preferred timing of antibiotic withdrawal. Significance of the intervention may be under-represented due to selection bias as those who were uncomfortable with treatment of uncomplicated CAP may not have answered the pre-intervention survey.

